# Why discrepancy between patient satisfaction and documentation of pain management?

**DOI:** 10.1186/1757-7241-23-S1-A47

**Published:** 2015-07-16

**Authors:** Marianne K Barylak, Christina Ø Rasmussen, Helle Ipsen

**Affiliations:** 1 Emergency Department, Nykøbing Falster Hospital, Nykøbing Falster, Denmark

## Introduction and purpose

Pain is one of the main causes leading to admission in the Emergency Department (ED) and has importance for patient satisfaction. Baseline LUP data of patient satisfaction has demonstrated that although patients are content, audit of the health care documentation of pain treatment seems insufficiently. Therefore, this study will investigate the difference between patient satisfaction, staff experience, and results from audit of the documentation of pain management in patient files. The hypothesis is that the documentation only describes a minor part of the efforts to treat the patient's pain.

## Materials and methodology

A qualitative investigation using a validated semi structured questionnaire of ED nurses' experience and attitude to pain management and documentation will be compared with documented care and treatment in patient files. Results will be analysed with qualitative analysis and presented as display. Baseline audit of documentation of pain treatment in 77 files and questionnaire with 5 staff nurses as indicator, followed by questionnaire of 20 nurses working 2 consecutive days.

## Results

The national LUP performed in 2011-2013 shows patient satisfaction of pain management 82-85%. Baseline audit shows that 85% of all patients in the ED have been asked about pain at arrival. 30% has got VAS/NRS score > 3, which demand a documented plan and follow up of the effectiveness of treatment (regional guideline) although audit shows it was only documented in less than 15% of the files. The questionnaire with 20 nurses identify that nurses have a holistic approach to pain treatment but the time factor and lack of system support is the main course of this weak documentation.

## Conclusion

The study has shown that nurses work with pain management in a way that might have importance for the patient satisfaction but it is not demonstrated in their documentation. Further studies will be performed involving the patients' ideas to identify and develop a pain management report.

